# The effect of community engagement on healthcare utilization and health insurance enrollment in Ghana: Results from a randomized experiment

**DOI:** 10.1002/hec.4556

**Published:** 2022-08-09

**Authors:** Stephen Kwasi Opoku Duku, Edward Nketiah‐Amponsah, Christine J. Fenenga, Wendy Janssens, Menno Pradhan

**Affiliations:** ^1^ Marie Stopes Ghana Accra Ghana; ^2^ Department of Economics University of Ghana Legon Ghana; ^3^ Department of Health Science University Medical Centre Groningen Groningen The Netherlands; ^4^ Vrije Universiteit (VU) Amsterdam and Amsterdam Institute of Global Health and Development (AIGHD) Amsterdam The Netherlands; ^5^ Vrije Universiteit (VU) Amsterdam Univeristy of Amsterdam and Amsterdam Institute of Global Health and Development (AIGHD) Amsterdam The Netherlands

**Keywords:** community participation, Ghana, health insurance, universal health coverage

## Abstract

Health insurance enrollment in many Sub‐Saharan African countries is low, even with highly subsidized premiums and exemptions for vulnerable populations. One possible explanation is low service quality, which results in a low valuation of health insurance. Using a randomized control trial in 64 primary health care facilities in Ghana, this study assesses the impact of a community engagement intervention designed to improve the quality of healthcare and health insurance services on households living nearby the facilities. Although the intervention improved the medical‐technical quality of health services, our results show that households' subjective perceptions of the quality of healthcare and insurance services did not increase. Nevertheless, the likelihood of illness and concomitant healthcare utilization reduced, and especially households who were not insured at baseline were more likely to enroll in health insurance. The results show that solely increasing the technical quality of care is not sufficient to increase households' subjective assessments of healthcare quality. Still, improving technical quality can directly contribute to health outcomes and further increase health insurance coverage, especially among the previously uninsured.

## 1 INTRODUCTION

While health shocks pose a major risk to the poor, the willingness to pay for health insurance remains low. Countries aiming for universal health coverage often have difficulties insuring informal sector workers who have to pay insurance premiums out‐of‐pocket because they are not poor enough to be covered under social assistance programs but they are also not covered by health insurance included in collective labor agreements. Research into the underlying causes in Nicaragua, Vietnam, the Philippines and Indonesia (Banerjee et al., 2019, 2021; Capuno et al., 2016; Wagstaff et al., 2016) found that interventions that reduced the price of insurance for a limited time in combination with providing assistance in the enrollment process increased enrollment by around 30% (Philippines and Indonesia); however, this was still insufficient to sustainably solve the problem of low uptake. Providing information on the importance of insurance had (almost) no effect in all cases.

One possible reason why it has proven so hard to raise enrollment is that interventions were mostly implemented in isolation from healthcare providers. Purchasing health insurance requires trust from the side of the buyer that quality health services will be provided at no cost. If service quality of contracted providers is low or if households feel that they will be treated worse when insured as compared to people who pay on the spot (Duku et al., 2018), people will be more inclined to go to non‐contracted providers, such as private clinics, drug vendors, or traditional healers, and pay out‐of‐pocket. Having health insurance then offers little added value and consequently demand for health insurance will remain low. Community health insurance schemes try to overcome this issue by organizing health insurance and care provision at a very local level, so that the commitment to finance is provided along with the commitment to deliver services (Devadasan et al., 2006).

In this paper we report on the impact of a Community Engagement (CE) intervention that aimed at increasing health insurance enrollment by improving the quality of healthcare services at the facilities contracted by the National Health Insurance Scheme (NHIS) in Ghana, as well as the quality of the insurance services themselves, such as the registration procedures. The intervention consisted of two rounds of assessments during which existing community groups or associations assessed the non‐technical quality^1^ of their nearest healthcare facility (Alhassan et al., 2015) as well as the services at their NHIS district office. Workshops were organized to report the findings of these quality assessments to the management of the healthcare facilities and NHIS district offices for them to address any quality gaps identified. Two rounds of quality assessments were done over a period of almost 1 year.

The experiment was unique in that it tried to address the problem of low uptake of health insurance by simultaneously addressing the problem of low perceived quality of healthcare services as well as the issues hindering insurance enrollment and renewal. Services were expected to improve as community members could directly communicate the quality gaps that they experienced and appeal to the pro‐social preferences of healthcare providers, alert NHIS officers of shortfalls in service delivery and apply informal pressure to deliver better services. Better services at the NHIS offices and contracted healthcare providers in turn would make health insurance more valuable. Moreover, participating community members were expected to raise awareness in the broader community on the importance of health insurance and the plans for improving healthcare quality and NHIS services, further increasing the demand for health insurance.

To test these predictions, we conducted a cluster Randomized Control Trial (RCT) in 16 NHIS districts in the Greater Accra and Western Regions of Ghana. Even with premium exemptions for vulnerable populations and highly subsidized premiums, enrollment coverage in the NHIS was low at 38% of the population at the time of the study (National Health Insurance Authority, 2014).^2^ We selected 64 NHIS‐contracted health facilities and randomly assigned half (32) to the intervention, and the remaining half (32) to the control group. Our impact analysis is based on household panel surveys covering a representative random sample of 1920 households living in the catchment areas of the selected healthcare facilities. The surveys were fielded in 2012 prior to intervention and in 2014, 3 months after completion of the intervention.

We find little evidence of impact on household quality perceptions. Although the intervention improved the medical‐technical quality of health services (Alhassan et al., 2015), households' subjective perceptions of the quality of healthcare services did not increase. There was also no significant effect on the perceived service quality of NHIS district offices. Nevertheless, the intervention significantly reduced the probability of illness and concomitant healthcare utilization on the extensive margin. For those who visited a health facility at least once, utilization increased – as expected if the intervention enhanced access to care. In addition, households in treatment communities who were uninsured at baseline significantly increased their enrollment in NHIS. We do not find a consistent impact on the renewal rates of insured households.

The experiment contributes to a literature that addresses the question of whether health services can be improved by increasing accountability toward clients through community participation. To our knowledge, our experiment is the first to explicitly involve health insurance providers in this process. So far, the evidence is mixed. One prominent, small‐scale (25 intervention clinics) study in Uganda (Bjorkman & Svensson, 2009) showed sustained effects of a report card type of intervention followed by a joint planning exercise with community groups and providers. The one‐off intervention, which lasted about 5 days, led to sustained improvements in mortality rates and utilization. A follow‐up study (Nyqvist et al., 2017), adding another 25 clinics to a new treatment arm, indicated that the provision of information on the baseline state of service delivery was key in the process. A treatment in which this information was not provided to community members did not yield impacts, and planning meetings tended to focus on external problems, rather than problems which could be solved by providers and communities.

The positive results from Uganda have not been replicated. A large‐scale replication study (Raffler et al., 2020) in Uganda (95 clinics in each of three treatment arms) found no effect on utilization and health outcomes of any of the treatment arms. The experiment was set up to test the importance of report card information versus creating an interface between providers and community members. Similarly, a report card study in India (Fabbri et al., 2019) (45 clinics in each of three treatment arms) also found no effected of any of the interventions on the targeted outcome of maternal care. That study was set up to test the importance of discussing the outcomes of the report card in provider groups, community groups or both. A very small study (10 treatment clinics) in Malawi, which included a joint community‐provider planning exercise, found mostly insignificant effects on service delivery (Gullo et al., 2017) and health governance (Gullo et al., 2018). They did find some positive effects on the outreach of community health workers.

Our findings confirm the growing evidence of a lack of effect of community participation interventions on the quality of healthcare as reported by households. However, as we highlight, there is a key difference between perceived and technical quality of care. The latter did improve as a result of the CE and positively affected health outcomes. We also contribute directly to the literature that investigates how to enhance the demand for health insurance in developing countries. We show that engaging communities in quality improvements of both healthcare and NHIS services can increase the enrollment in health insurance among the previously uninsured. To our knowledge, this has not been shown before.

The rest of the paper is organized as follows. The next section describes the study design, the intervention and the econometric methodology of the study. Subsequently, we present the results of our data analysis. We then discuss the results, followed by conclusions with policy recommendations.

## 2 RESEARCH METHODOLOGY

### 2.1 Research setting and design

The study was conducted in the Greater Accra and the Western Regions of Ghana. These Regions are situated along the Eastern and Western coast of Ghana respectively with contrasting differences in rural and urban populations. The Greater Accra Region that includes the capital city of Ghana, has a largely urban population of about 4 million, accounting for 16.3% of the national population while the Western Region has a predominantly rural population of about two million representing 9.6% of the national population (GSS, 2010). These two Regions were purposively selected to provide a rural/urban balance as well as a socio‐economic structure representative of Ghana.

The project was implemented as a RCT based on a three‐stage stratified sampling procedure. In the first stage, 16 NHIS district offices (eight from each region) were randomly sampled. Next, four NHIS‐accredited primary healthcare facilities in the catchment area of each of the NHIS district offices were randomly selected to obtain a total of 64 healthcare facilities, 32 each in Greater Accra and Western Regions, respectively. The 64 facilities constitute 5% of the approximately 1180 accredited clinics/health centers in Ghana, or about 20% of the total number of accredited health facilities in the two study Regions.

In the third stage, 30 households were randomly sampled from the catchment area of each of the selected health facilities to make a total sample of 1920 households. This sample size is sufficient to detect a minimum effect size of a 5.0% point increase in active enrollment in NHIS from a baseline enrollment of 38% (see Appendix 1 for detailed power calculations).

Random assignment was blocked at the level of the NHIS district office, such that in each district two health facilities were assigned to the intervention group and two health facilities were assigned to the control group. In each district, the names of the four health facilities were written on pieces of paper. Subsequently, for each district at a time, two ballots (representing health facilities) were randomly picked without replacement to receive the intervention. Thus, out of the 64 health facilities, 32 each were then randomly allocated into the control and intervention groups of the project.

Two rounds of household surveys were conducted. The baseline household survey collected data on all 1920 households in 2012 prior to the start of the intervention. The follow‐up survey was conducted in 2014, 3 months after the completion of the intervention among the same households who were interviewed at the baseline. Structured household questionnaires were developed to collect data on demographic and socio‐economic characteristics, health status and healthcare utilization, as well as health insurance enrollment of all household members at the baseline and follow‐up surveys. In addition, household heads were interviewed on their perceptions of the quality of healthcare and of NHIS services in both rounds of the survey. The questionnaires were administered either in English or in one of the two local languages Fante and Ga of the Western and Greater Accra Regions, respectively, by experienced and trained interviewers who were fluent in English and the local languages.

### 2.2 Description of the community engagement intervention

The CE intervention was implemented for almost 1 year (from June 2013 to March 2014) in the intervention communities. It was developed based on a comprehensive, interdisciplinary study of quality‐related obstacles to enrollment in health insurance in the study areas, as documented in (Fenenga et al., 2015), yielding the following theory of change: If the healthcare services at the (public) clinics contracted on the insurance scheme are of low quality, people will not be inclined to buy insurance that covers these services. Instead, they will prefer alternatives such as private clinics, traditional healers, or patent medicine vendors; insurance offers limited perceived added value. In addition, low quality of the insurance services themselves (such as cumbersome enrollment procedures, long waiting times before receiving the insurance card, or a lack of complaint handling) will add to the costs of enrollment and further reduce the demand for health insurance.

The CE intervention addresses these problems by engaging communities into healthcare and insurance service quality improvements. Community feedback sessions on perceived quality gaps with both healthcare providers and insurance officers give communities voice, and enhance communication and understanding between patients/enrollees, providers, and the insurer. Repeated sessions will enable healthcare facilities and insurance offices to address highlighted issues and improve their services. As a result, we expect the intervention to have a positive effect on health insurance enrollment because it increases value for money – either directly when insurance service delivery improves or indirectly when the quality of covered healthcare treatments improves.

The intervention was implemented using existing community groups and associations to assess the quality of healthcare and of NHIS services in the selected facilities and NHIS district offices.^3^ The implementation involved six steps, as follows^4^:

Step 1 The first step involved the recruitment and training of facilitators and the identification of existing community groups/associations in the intervention communities. One facilitator was assigned to each of the community groups. Eligibility criteria for selection of community groups included: (i) documented evidence of routine meetings (at least four times a year), (ii) regular meeting venue, (iii) clear leadership structure, (iv) non‐partisan and (v) active membership not less than an intuitive number of ten. These criteria were meant to ensure the groups were active in their communities and represented a reasonable cross‐section of community opinion. Where more than two community groups around the catchment area of a health facility met these eligibility criteria, simple random sampling was done to select two groups. The groups were made to understand that participating in the intervention activity was a noble role that their group could play in ensuring that the services provided by the health facility and the NHIS office would be of better quality, which they willingly agreed to do. Participation was voluntary, and participants received no compensation for their participation aside from travel expenses.

Even though the types of community groups varied based on core activities, these features did not systematically relate to particular features of the sampled clinics. The community groups recruited comprised 22 religious/faith‐based groups, eight traders' groups, one widows' group, three community volunteer groups, three musician groups, five artisans' groups and 11 youth groups. Average group size during the assessment was 29 members (SD = 20). More than half of the groups were female dominated, 13 were male dominated, two were all males, five were all females, and one was a balanced group of both males and females. Approximately 56% of the groups were a combination of literates and illiterates, 23% were mainly literates, and 21% mainly illiterates. In terms of age, 35% had predominantly youthful members (18–30 years), and the remaining 65% had predominantly older members (31+ years). The details of the selected community groups/association characteristics can be found in Alhassan et al. (2015).

Step 2 The second engagement step entailed a first round of community group assessments of (i) the (non‐technical) quality of healthcare services, and (ii) the quality of NHIS services, based on group members' most recent (at most 6 months) experience with the particular health facility in their community, and the nearest NHIS district office, respectively. The assessment categories were developed during several joint workshop sessions with the three key stakeholders – healthcare clients, healthcare providers and the health insurer (Fenenga et al., 2016).

The proxies used to guide community members during their assessment of the quality of healthcare services were: (1) staff attitude, (2) punctuality to work, (3) client waiting time, (4) queuing system, (5) availability of drugs, (6) information provision to clients, (7) equal treatment for insured and uninsured clients, (8) complaint system for clients, (9) client‐provider communication, and (10) net promoter score (NPS). The NPS is an indicator used to determine the possibility of clients recommending the health facility to others (e.g., relative, friend or co‐worker) based on their personal experiences of the quality of health service delivery.

The proxies for the assessment of services delivered by the NHIS district office included: (1) attitude of staff, (2) punctuality of staff, (3) information provision to clients, (4) NHIS enrollment/renewal process, (5) delivering what the NHIS promised to clients, (6) complaint filing system for clients and (7) NPS.

During the CE sessions, group members rated performance of their nearest health facility and their NHIS district office on these service quality proxies on a five‐point Likert scale from 1 = “Very disappointing” to 5 = “Very satisfactory”. The group assessments were conducted in the communities to avoid possible bias and client intimidation when done at the health facility or NHIS office. Anonymity of group members was assured by reporting group perception ratings without members' personal details. The average meeting duration per group was 41 min (SD = 13.8) (Alhassan et al., 2015).

Step 3 The third implementation step involved regional‐level validation and feedback sessions in the two regional capitals of the study regions to disseminate the group assessment findings with facility heads, clients and NHIS district office representatives. This platform offered the healthcare providers and NHIS district officers the opportunity to recognize and accept gaps in non‐technical service quality and agree on quality improvement plans with timelines and responsible persons.

Step 4 During the fourth step (3 months after the validation and feedback sessions), facilitators followed‐up with the healthcare providers and NHIS district officers to ascertain whether they were implementing the agreed action plans toward quality improvement or not.

Step 5 The fifth step – again 3 months later, and approximately 6 months after the first round of community group assessment – involved the second round of community group assessments of healthcare and NHIS services quality based on group members' most recent experience (past 3 months) with the health facility in their community, and their NHIS district office, respectively. The same quality proxies used in the first round of assessment was used in the second round. The second round of assessment was to ascertain whether the efforts put in by the healthcare providers and the NHIS district offices had resulted in any change in group members' perceptions about the service quality. The average quality assessments improved across all dimensions from the first to the second round (Alhassan et al., 2016).

Step 6 The last step involved two joint stakeholder validation and award ceremonies in the regional capitals of the project regions to reward the best performing health facilities after the second round of community assessment. A citation plaque of honor and a token financial incentive of GHC 1000.00 (Ghanaian Cedis), approximately US$ 280.0 was awarded to one best performing healthcare facility per district. The selection was based on the level of efforts in addressing the identified quality gaps as adjudged by the community groups/associations during the second round of assessment to encourage competition among peers toward quality improvement. No performance reward was given to NHIS district offices that performed well in addressing the identified quality gaps.

Three months after the completion of the CE intervention, the follow‐up household survey was conducted in the same households that were interviewed at baseline to assess whether the intervention helped to improve the perception of non‐technical quality of healthcare and NHIS services in the general community population.

We emphasize that the households in the survey were not necessarily participants in the intervention. Nevertheless, at populations of approximately 500 inhabitants, an average of about 60 participants per community implied a coverage of approximately 10 percent of the population, and a larger fraction of households. In addition, the group leaders generally announced the group meetings at the mosque on Friday, in Church on Sunday or through market queens on market days. We therefore expect that the intervention reached a sizable section of community members and not just the group/association participants, although we did not collect data to quantify reach of the announcements.

**FIGURE 1:**
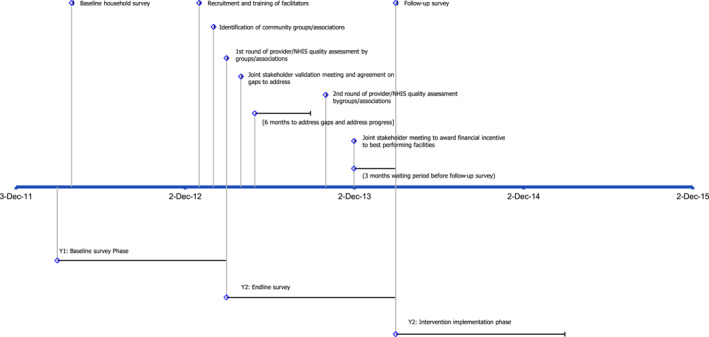
Timeline of intervention implementation activities

Figure 1 presents the timelines of activities for the implementation of the intervention and data collection.

### 2.3 Ethical review

Moved to title page to enable blind review.

### 2.4 Survey response rate

**FIGURE 2:**
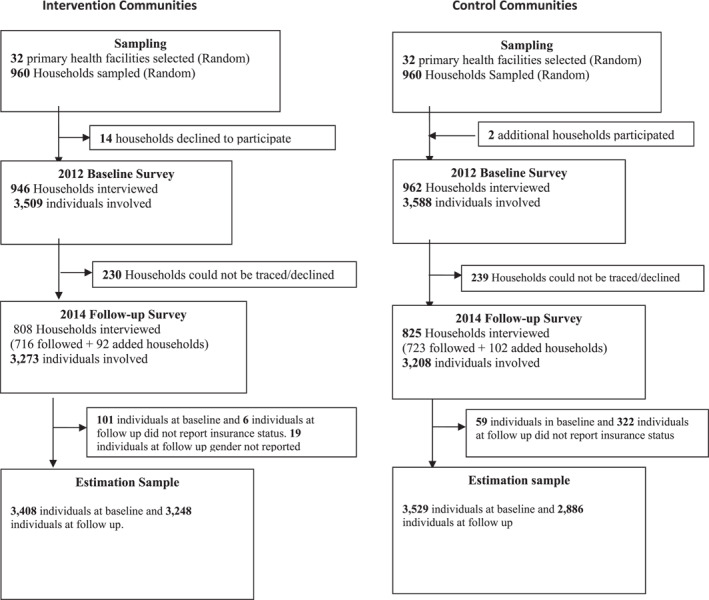
Participation in the 2012 and 2014 surveys

Out of the sample of 1920 households, 1908 were interviewed at baseline involving 7097 individuals (see Figure 2). Fourteen households in the intervention communities declined to participate in the baseline survey leaving 946 households representing a 98.5% response rate, involving 3509 individuals. In the control communities, two additional households were sampled which brought the total number of control households interviewed to 962, involving 3588 individuals. During the follow‐up survey, out of the 1908 households interviewed at baseline, 1439 were traced and interviewed, representing a 75.4% overall tracking rate, involving 5451 individuals. This represents our balanced sample of individuals who participated in the baseline and the follow‐up surveys. In the intervention communities at follow‐up, 230 households could either not be traced or declined to participate. In the control communities, 239 households could either not be traced or declined to participate. However, 92 and 102 additional households in the intervention and control communities, respectively, were sampled to bring the total number of households interviewed at follow‐up to 1633 (808 intervention and 825 control) and total individuals interviewed at follow‐up to 6481.

### 2.5 Outcome measures

The primary outcomes of interest are perceptions of non‐technical quality of healthcare and NHIS services, frequency of reported illness, frequency of health facility visits and voluntary enrollment in the NHIS. The frequency of reported illness was used as a proxy measure of the adoption of behaviors that promote good health and prevent illness. Frequency of health facility visits was used as a proxy measure for formal healthcare utilization.

Perception of non‐technical quality of services^5^ was measured at the level of the household head, in line with the assessments during the CE intervention. Respondents were to indicate their level of satisfaction or agreement on a Likert scale from 1 representing strong disagreement/dissatisfaction to 5 representing strong agreement/satisfaction to 10 statements about the quality of healthcare and nine statements about the quality of NHIS services. These statements were subsequently summarized using factor analysis into four continuous factor scores each, based on factor loadings and Cronbach's alpha greater than 0.5. Factor means were normalized at zero with standard deviation equal to one, and used as the summary measure in the analysis. Appendix Table A1 presents the underlying perception statements, their means, factor loadings and Cronbach's alphas of the factors.

The four factors for perception of healthcare quality are: (1) Complaint lodging, handling and feedback; (2) Respect, compassion and friendliness of health staff; (3) Adequacy of Information provision and service delivery; and (4) Satisfaction with waiting time. The four factors for NHIS service quality are: (1) Information, service delivery and NHIS benefit; (2) ID card production and distribution; (3) Registration fee and annual premium; and (4) Office location and opening hours. Two additional indicators, “Overall healthcare quality” and “Overall NHIS service quality”, were computed as the average of the factor scores for healthcare and NHIS quality, respectively.

The frequency of illness was measured with a count variable indicating the number of times an individual reported ill within the 6 months prior to the survey. Frequency of (outpatient) health facility visits in the 6 months prior to the survey was measured with a count variable indicating the number of visits to a modern orthodox healthcare provider (health center/clinic, maternity home, private doctor/nurse practice or hospital). The frequency of health facility visits was unconditional on illness such that people who never reported ill were included with a zero (0).

Enrollment in the NHIS was measured at the individual level, as some households may choose to enroll only some family members. It was defined as: (i) the voluntary payment of registration fee and insurance premium to acquire the insurance membership card for informal sector employees; or (ii) the payment of registration fee to acquire the health insurance membership card for formal sector employees, or (iii) the issuance of a health insurance membership card to an individual under any of the categories of the NHIS premium exemption policy (children under 18 years, people 70 years and above, Social Security and National Insurance Trust pensioners, pregnant women, or Indigents and Livelihood Empowerment Against Poverty beneficiaries). Insurance enrollment was measured at the time of the interview with a dichotomous variable equal to one if the individual was insured, and 0 otherwise.

### 2.6 Estimation strategy

Bivariate analysis (Pearson Chi‐square test for categorical variables and *t*‐test for continuous variables) correcting for clustering at the health facility level is done first to compare the differences in respondents' characteristics between the control and intervention communities at the baseline.

We next estimate a series of ordinary least square (OLS) regressions of the form:

(1) $$Y_{igt}=\beta_{1}J_{g}+\beta_{2}t+\beta_{3}\left(J_{g}.t\right)+\beta_{k}X_{igt}+\gamma_{g}S_{g}+\varepsilon_{igt}$$

Where ***Y_igt_*** is the outcome of interest (perceived healthcare quality, perceived NHIS services quality) for individual ***i*** living in the catchment area of facility ***g*** at time ***t. J***_***g***_ is a dummy variable which equals 1 for the treatment group and 0 for the control group, ***t*** indexes time periods (1 = post‐treatment, 0 = pre‐treatment), ***X***_***igt***_ is a set of individual characteristics and ***S***_***g***_ are NHIS district indicators (the randomization strata) since randomization was blocked at the level of the NHIS district. ***ε***_***igt***_ is the error term, corrected for clustering at the health facility level. Thus, Equation (1) follows a difference‐in‐differences specification, where *β*_1_ denotes the pre‐intervention difference between control and treatment groups, *β*_2_ the common time trend, and *β*_3_ is the treatment effect. Our preferred estimate uses this model for the unbalanced sample. As robustness checks, in the appendix, we also provide the estimates where we omit the controls ***X***_***igt***_, and one where we use only the balanced sample (with controls).

For outcome variables which indicate classes rather than values, we use limited dependent variable models. For health insurance enrollment, we use a Probit specification, where the latent variable is modeled as in (1). For the frequency of illness and health care utilization, which are count variables, we use a hurdle model. Following Deb and Norton (2018), we also considered a Poisson and Negative Binomial model rather than a hurdle model, and on the basis of the Bayesian information criterion decided on the final specification. The hurdle model uses a Logit model to model whether the outcome variable is zero or positive, and a Truncated Poisson model for positive outcomes. For each model, the right‐hand side variables are included in the same way as in Equation (1). As the estimated coefficients of limited dependent variable models are difficult to interpret, we also present marginal effects, which indicate the average predicted increase in the outcome at endline as a result of treatment.

## 3 RESULT

### 3.1 Characteristics and balance of the baseline sample

The demographic characteristics of respondents at baseline are reported in Table 1 Panel A. The average respondent was 26 years old, with the majority (54%) being female and 51% living in rural communities. The average household consisted of five people, and most (89%) were Christians. Table 1 Panel B presents the socio‐economic characteristics of respondents at baseline. On average, 49% had completed primary education or above, and 43% had been in gainful employment in the last 12 months. The average annual household expenditure was GH₵3972.09.^6^ One third (31%) of respondents had been sick at least once, and 38% had visited a facility for healthcare services within the last 6 months prior to the baseline survey (Table 1 Panel C). Less than half (42%) of respondents were insured with the NHIS at the time of the baseline survey (Panel D of Table 1). Panels E and F of Table 1 present the average perceptions of household heads on the five indicators of healthcare quality and NHIS service quality, respectively.

**TABLE 1:** Comparison of the intervention and control group at baseline

	2012 baseline survey						
	# Obs.	Mean	Std. Err.	Inter‐vention	Control	Difference	*p*‐value
	**1**	**2**	**3**	**4**	**5**	**6**	**7**
Panel A. Demographic characteristics (N = 6937)							
Average age	6937	26.19	0.412	26.45	25.95	0.494	0.551
% females	6937	54.43	0.680	54.93	53.95	0.977	0.479
% living in rural communities	6937	50.97	6.381	51.85	50.13	1.721	0.893
Average household size	6937	4.84	0.081	4.84	4.83	0.010	0.950
% Christians	6937	89.48	0.991	89.23	89.71	−0.483	0.808
Panel B. Socio‐Economic characteristics (N = 6937)							
% primary education & above	6937	48.97	1.583	49.71	48.26	1.449	0.650
% employed	6937	42.69	0.594	43.05	42.36	0.683	0.567
Average annual household expenditure (GH₵)	6803	3972	447	4581	3393	1188	0.191
Panel C. Health Status and healthcare utilization (N = 6937)							
% Sick in last 6 months	6937	31.01	1.754	29.64	32.33	−2.696	0.443
% Visiting health facility in last 6 months	6937	38.47	1.835	38.88	38.08	0.795	0.829
Panel D. Insurance enrollment rate							
% currently insured	6937	41.98	1.594	44.54	39.50	5.04	0.111
Panel E. Household head's perception of healthcare quality (N = 1908)							
Average perception on overall healthcare quality	1908	0.000	0.028	0.007	−0.007	0.014	0.797
Average perception on complaint lodging, handling & feedback	1849	0.000	0.037	0.022	−0.021	0.043	0.557
Average perception on respect, compassion & friendliness	1886	0.000	0.032	0.001	−0.001	0.001	0.983
Average perception on information & service delivery	1868	0.000	0.029	−0.006	0.006	−0.012	0.845
Average perception on waiting time	1790	0.000	0.037	0.014	−0.014	0.028	0.710
Panel F. Household Head's perception of NHIS service quality (N = 1908)							
Average perception on overall NHIS quality	1908	0.000	0.017	−0.023	0.022	−0.045	0.179
Average perception on information & service provision	1882	0.000	0.035	−0.049	0.048	−0.097	0.162
Average perception on ID card production & waiting time	1880	0.000	0.026	−0.032	0.031	−0.063	0.229
Average perception on registration fees & annual premium	1887	0.000	0.029	−0.021	0.021	−0.042	0.470
Average perception on office location & opening hours	1883	0.000	0.032	0.009	−0.009	0.019	0.764

*Note*: Full (unbalanced) sample. The sample sizes for Panels E and F are smaller because the perception questions were posed only to household heads. P‐values and standard errors are robust and corrected for clustering at the health facility level.

Abbreviation: NHIS, National Health Insurance Scheme.

*p < 0.10, ** *p* < 0.05, *** *p* < 0.01.

*Source*: COHEiSION Project data.

Table 1 Columns (4)‐(7) compare respondent characteristics in the intervention and control communities at baseline. The differences were small and statistically insignificant. This underscores the fact that the randomization resulted in two comparable samples such that the control group represents an adequate counterfactual for the intervention group. When looking at the balanced rather than the full sample, the differences in baseline characteristics were also statistically insignificant (see Appendix Table A2). The attrition analysis further indicates that receiving the intervention did not significantly predict attrition (see Appendix Table A3).

### 3.2 Impact of community engagement on perceived quality of healthcare and NHIS services

The impact of the CE intervention on household heads' perceptions of non‐technical healthcare quality is presented in Table 2 for the full sample with control variables. The results for the full sample without controls, or for the balanced sample are very similar (see Appendix Tables A4 and A5, respectively. The interventions did not have a significant impact on any of the four factor scores of perception of healthcare quality, nor on overall healthcare quality. Signs of the impact coefficients are negative, but their sizes are small throughout, and standard errors are large.

**TABLE 2:** Impact of community engagement on household heads' perception of healthcare quality

	Overall healthcare quality	Complaint lodging & feedback	Respect & friendliness of staff	Information & service delivery	Waiting time
	(1)	(2)	(3)	(4)	(5)
Year	0.024	0.039	0.021	0.022	0.013
	(0.075)	(0.099)	(0.078)	(0.072)	(0.093)
Treatment group	0.017	0.050	0.002	−0.009	0.028
	(0.047)	(0.068)	(0.052)	(0.051)	(0.061)
Treatment group * year (=Impact of CE)	−0.037	−0.073	−0.027	−0.026	−0.022
	(0.096)	(0.127)	(0.102)	(0.098)	(0.129)
Controls	Yes	Yes	Yes	Yes	Yes
Strata fixed effects	Yes	Yes	Yes	Yes	Yes
Observations	3515	3456	3493	3475	3397

*Note*: Based on the full (unbalanced) sample. Estimates are based on OLS regressions. Standard errors are in parentheses, robust and corrected for clustering at the health facility level. All equations include randomization strata fixed effects and sex, religion, household size, employment status, educational level as control variables.

Abbreviations: CE, Community Engagement intervention; OLS, ordinary least square.

*p < 0.10, **p < 0.05, ***p < 0.01.

The impact of the intervention on perceptions of NHIS service quality is presented in Table 3 for the full sample with controls. Again, results for the full sample without controls, and for the balanced sample are very similar (Appendix Tables A6 and A7, respectively). The results show that the intervention did not have a significant impact on any of the indicators of perception of NHIS service quality.

**TABLE 3:** Impact of community engagement on household heads' perception of national health insurance scheme (NHIS) service quality

	Overall insurance services quality	Information & service delivery	ID card production & distribution	Registration fees & annual premium	Office location & opening hours
	(1)	(2)	(3)	(4)	(5)
Year	0.001	0.007	0.030	−0.042	0.007
	(0.055)	(0.094)	(0.084)	(0.044)	(0.074)
Treatment group	−0.046	−0.098	−0.064	−0.044	0.019
	(0.037)	(0.065)	(0.050)	(0.050)	(0.062)
Treatment group * year (=Impact of CE)	0.005	−0.003	−0.052	0.086	−0.007
	(0.075)	(0.124)	(0.109)	(0.065)	(0.115)
Controls	Yes	Yes	Yes	Yes	Yes
Strata fixed effects	Yes	Yes	Yes	Yes	Yes
N	3515	3489	3487	3494	3490

*Note*: Based on the full (unbalanced) sample. Estimates are based on OLS regressions. Standard errors are in parentheses, robust and corrected for clustering at the health facility level. All equations include randomization strata fixed effects and sex, religion, household size, employment status, educational level as control variables.

Abbreviations: CE, Community Engagement intervention; OLS, ordinary least square.

*p < 0.10, **p < 0.05, ***p < 0.01.

### 3.3 Impact of community engagement on service utilization and health insurance enrollment

Table 4 shows the impacts of the intervention on the likelihood and frequency of illness, that is, on the extensive and intensive margins, respectively, in Columns (1) and (2). It shows the impact on the likelihood and frequency of health facility visits in Columns (3) and (4), respectively. All estimations are done on the full sample with controls.

**TABLE 4:** Impact of community engagement (CE) on frequency of illness and health facility visits

Dependent variable	Frequency of illness		Frequency of health facility visits	
	Extensive margin	Intensive margin	Extensive margin	Intensive margin
	Logit	Truncated Poisson model	Logit	Truncated Poisson model
	(1)	(2)	(3)	(4)
Year	1.519***	0.145	−0.351***	0.701***
	(0.126)	(0.175)	(0.100)	(0.221)
Treatment group	−0.060	−0.096	0.125	−0.380
	(0.121)	(0.166)	(0.100)	(0.256)
Treatment group * year (=Impact of CE)	−0.356*	0.017	−0.283*	0.534*
	(0.190)	(0.182)	(0.157)	(0.274)
Controls	Yes	Yes	Yes	Yes
Strata fixed effects	Yes	Yes	Yes	Yes
*N*	13,071	4590	13,071	3646
Marginal effect of the impact of CE	−0.076*	0.007	−0.048*	0.323*
	(0.041)	(0.076)	(0.027)	(0.176)

*Note*: Based on the full (unbalanced) sample. Estimates are based on a hurdle model with a logit in the first stage (extensive margin) and a truncated Poisson model in the second stage (intensive margin). Standard errors are in parentheses, robust and corrected for clustering at the health facility level. All equations include randomization strata fixed effects and sex, religion, household size, employment status, educational level as control variables.

Abbreviation: CE, Community Engagement intervention.

*p < 0.10, **p < 0.05, ***p < 0.01.

The time indicators show that overall, the prevalence of illness increased substantially from baseline to follow‐up. Households living in treatment areas however experienced a lower increase then households in the control areas. Column (1) shows that the intervention resulted in a significant reduction in the probability of illness of 7.6% points. Findings are of a similar order of magnitude when controls are excluded, and for the balanced sample, showing a 8.4 and 6.9% points decrease, although the latter is less precisely estimated and not significant at conventional levels (Appendix Table A8 and A9). Conditional on being ill, the intervention did not reduce the frequency of illness at an insignificant marginal effect of 0.007% points.

Likewise, the intervention had a significant negative impact on the likelihood of a health facility visit, as shown in Column (3), both with and without controls (Appendix Table A8) and for the full as well as the balanced sample (Appendix Table A9). The probability of conducting at least one health visit decreased for all households in the study area, as shown by the coefficient on the Year variable, but it decreased even further with 4.8% points for the households in the treatment group – in line with the reduction in the probability of illness. Conditional on visiting a health center at least once, program impact on the frequency of visits was significantly positive, indicating higher healthcare utilization in treatment areas.

The impact of the intervention on health insurance enrollment is investigated in Table 5 with controls (and in Appendix Table A10 without controls). Overall, we do not find a significant effect of the CE intervention on enrollment, neither on the full sample nor the balanced sample in Columns (1) and (2), respectively. Columns (3) and (4) split the balanced sample in households that were uninsured and insured at baseline, respectively. Column (3) shows that the intervention resulted in a significant 5.9% points increase in health insurance enrollment among the uninsured at baseline for the preferred estimation with controls, and a 10.6% points increase when controls are excluded (Appendix Table A10). Impact on the insured at baseline – which captures the probability of renewal – is not statistically significant in Column (4), although the estimation without controls in Appendix Table A10 yields a significant marginal increase of 7.4% points for the insured as well.

**TABLE 5:** Impact of community engagement (CE) on insurance enrollment

	Insured	Insured	Insured at endline	Insured at endline
			Balanced sample	Balanced sample
Dependent variable	Unbalanced sample	Balanced sample	Uninsured at baseline	Insured at baseline
Sample	(1)	(2)	(3)	(4)
Year	−0.053	−0.089		
	(0.071)	(0.073)		
Treatment group	0.116*	0.128*		
	(0.064)	(0.068)		
Impact of CE (treatment group * year)	0.041	0.074	0.202*	0.124
	(0.088)	(0.089)	(0.114)	(0.122)
Controls	Yes	Yes	Yes	Yes
Strata fixed effects	Yes	Yes	Yes	Yes
N	13,071	10,483	2923	2112
Marginal effect (impact of CE)	0.015	0.028	0.059*	0.044
	(0.033)	(0.033)	(0.033)	(0.043)

*Note*: Column 1 is based on the full (unbalanced) sample, Column 2 on the balanced sample. Columns 3 and 4 are based the balanced sample, for the subsets of households that were uninsured and insured, respectively, at baseline. Estimates are based on probit models. Columns 3 and 4 use only endline data in the estimation and estimate insurance status at endline as a function of treatment. Standard errors are in parentheses, robust and corrected for clustering at the health facility level. All equations include randomization strata fixed effects and sex, religion, household size, employment status, educational level as control variables.

Abbreviation: CE, Community Engagement intervention.

*p < 0.10, **p < 0.05, ***p < 0.01.

Appendix Tables A11 and A12 further investigate heterogeneous impacts on health outcomes and healthcare utilization by baseline insurance status. The decrease in the probability of at least one illness and one healthcare visit is most pronounced for those households that were not insured at the start of the intervention (that had most to gain). Conditional on utilizing services at least once, the increase in the frequency of healthcare visits is strongest for households that were already insured.

## 4 DISCUSSION

The intervention built on CE in order to identify service delivery gaps in greater depth and detail, feeding back these gaps to healthcare providers and NHIS officers in order to stimulate a holistic appreciation of clients' concerns. This was expected to motivate providers and NHIS officers to implement strategies toward the provision of client‐centered services, which in turn would improve clients' perceptions of the quality of these services, increase utilization of health facilities at early stages of ill health and enhance enrollment in health insurance to avoid out‐of‐pocket payments.

Despite the prior expectations, the intervention did not result in significant improvements of client perceptions – neither in terms of quality of healthcare treatment nor quality of health insurance services. This stands in contrast to the findings from companion papers, which have shown that the intervention resulted in a significant improvement in the medical‐technical quality of care (Alhassan, Nketiah‐Amponsah, et al., 2015; Alhassan et al., 2019). The community groups observed this improvement in quality as well, as witnessed by their assessment reports (Alhassan et al., 2016). In support of these findings, Fenenga et al. (2018) showed qualitatively that CE indeed led to improved collaboration and communication among clients, healthcare providers and insurers.

Several hypotheses might explain why the CE intervention failed to impact significantly on clients' quality perceptions. On the one hand, it is possible that service providers intended to implement quality improvement strategies. However, the intensity and degree of these improvements may have been hindered by limited availability of health system structures and lack of funds. If healthcare and insurance providers lack financial resources to train staff on customer relations, for instance, then improvement in quality may be minimal and hence too subtle and limited to positively impact on the perceptions of service quality among households who did not participate in the community assessments themselves.

Another potential explanation is that quality did improve in specific domains, but that the changes were not picked up by our household measurement instruments. This explanation is supported by (Alhassan, Duku, et al., 2015), who find that perception of good quality healthcare among providers and patients often do not overlap. Whereas providers focus more on medical‐technical aspects of quality, patients value “softer” aspects such as staff attitudes and waiting times. This explanation might hence account for at least part of the insignificant findings on households' quality perceptions in the broader community.

Nevertheless, and even though households did not perceive a quality improvement, our results show that 2 years after baseline, health indicators in the treatment communities had improved significantly compared to the control group. The intervention resulted in a systematic reduction in the reported likelihood of illness and the concomitant likelihood of visiting a health facility at least once. The significant decrease in the probability of illness could potentially be attributed to people seeking prompt healthcare (because they had become more appreciative of the technical quality of care, or because they were now financially protected through insurance uptake (Asibey & Agyemang, 2017)), or because the care provided was of better quality (Adhvaryu & Nyshadham, 2015). While at the health facility, they may also have received additional preventive services or ensured that other health complaints were addressed. Finally, it is possible that the intervention made people more aware of the risk of infections and illness, which made them invest more in preventive health. Any of these pathways may have improved their future health, as picked up in the endline survey. Further research is needed to shed additional light on these potential mechanisms.

Despite the evidence being suggestive of improved health status, we do not find a significant change in the *frequency* of illness for those who were ill. This suggests that the program operated mostly on the extensive margin, benefiting the subset of households on the tipping point who turned from a bad to a good health status. Those who remained in poor health and visited a provider at least once were more likely to seek care in treatment versus control communities, suggesting a positive impact of health insurance on access to care. This effect was concentrated on households that were already insured at baseline.

Indirect costs of care, such as transportation and opportunity cost of time spent at the facility, have been documented as important barriers to healthcare utilization for the insured and the uninsured alike (Ettling & Shepard, 1991; McIntyre et al., 2006; Sauerborn, Nougtara, Hien, & Diesfeld, 1996). If indirect costs of care are unaffordable, improving the quality of care alone may not be sufficient to increase healthcare utilization, even in combination with insurance. The lack of significant findings on utilization for the previously uninsured could also be due to two counterbalancing effects. On the one hand, improved health status as indicated by the reduced prevalence of illnesses may have decreased the need for health facility visits, while on the other hand, improved quality of care and financial protection may have led to a greater demand for care (Asibey & Agyemang, 2017; Oberoi et al., 2016).

Impact on health insurance enrollment was not significant for the overall population. However, the intervention significantly increased the likelihood of enrollment especially for the uninsured at baseline. This suggests that engaging community members in the assessment and improvement of healthcare and insurance services may increase households' willingness to enroll, even if they do not directly see those efforts translated into perceived quality improvements. The enhanced health status of individuals in treatment communities might have added to this effect. Apparently, those who were already enrolled at baseline did not need this additional motivation to renew their insurance.

Still, a substantial portion of the study population remained uninsured. The fact that the intervention did not impact positively on perceptions of NHIS service quality suggests that many of the quality areas of concern to clients did not receive adequate attention or that improvements were too limited to strongly affect uptake. In addition, a previous study on insurance enrollment among Ghanaian adults revealed that socio‐economic factors such as being employed or being in the higher income brackets significantly predict enrollment (van der Wielen et al., 2018). Other major barriers to NHIS enrollment have been documented to include delays in re‐imbursements and NHIS registration/renewal process, ID card production, stock‐outs of drugs, inconvenience of NHIS office location and inadequate information on the benefit package (Jehu‐Appiah et al., 2011). The problem of delayed reimbursement had become incessant during the period of the intervention with intermittent withdrawal of services by the Christian Health Association of Ghana (CHAG) during which insured clients were “compelled” to pay out‐of‐pocket. The lack of impact of the intervention may also be partly due to the short‐term (12 months) implementation period.

Finally, the project team could not fully control the enrollment activities conducted by the NHIS and other NGOs in some of the project communities. In early 2014 – just about 2 weeks before the endline survey, the NHIA with the support of other NGOs, embarked on an NHIS information and sensitization campaign, including free enrollment of poor and vulnerable people in three interventions and three control communities in the Western Region (Citifm, 2015). This activity, which educated people in both the treatment and the control communities and enrolled indigents and poor people for free might have suppressed the overall effect of the engagement intervention on insurance enrollment. However, excluding these six intervention and control communities from the analysis does not affect the sign or significance of the impact results on health insurance enrollment.

## 5 CONCLUSION

This paper evaluated the impact of a CE intervention on households' perception of non‐technical quality of healthcare and NHIS services, as well as the probability and frequency of illness, healthcare utilization and health insurance enrollment in two Regions in Ghana. We found that the intervention did not have a measurable impact on households' subjective perceptions of service quality, although CE did improve the technical quality of care as shown in other studies. Nevertheless, as a result of the improved quality, the probability of illness among the population in treatment areas decreased by 7.6% points, suggesting an overall improvement in their health status. The engagement of communities also significantly increased healthcare utilization with 0.323 visits for those who visited a care provider at least once, which is suggestive of increased access to care. Health insurance enrollment increased especially among households that were not enrolled at baseline.

## CONFLICT OF INTEREST

All authors report financial support from The Netherlands government through the Ministry of Foreign Affairs and the Science for Global Development (WOTRO) which is a division of the Netherlands Organization for Scientific Research (NWO), under the Global Health Policy and Systems Research (GHPHSR) program (Project No. W07.45.104.00), during the conduct of the study.

## ETHICS STATEMENT

Ethical clearance for the study was obtained from the Ghana Health Service (GHS) Ethical Review Committee (ERC) [clearance numbers: GHS‐ERC: 18/5/11 and GHS‐ERC 08/5/11]. Informed consent was obtained from individual respondents in the communities for the baseline and follow‐up surveys, and also from community group/association leadership for the CE assessments. Literate respondents provided written informed consent while illiterate respondents thumb‐printed the informed consent form before participating in the study.

## Data Availability

Data available on request from the authors. We will make replication files available in a public location if the paper once the paper is accepted for publication.

## ENDNOTE

^1^
The quality of healthcare is often categorized into technical (or medical‐technical) and non‐technical (perceive) quality, with the former referring to structured processes and professionally defined practices and protocols of care, while the latter focuses on perceptions, experiences and satisfaction with the service delivery processes (Alhassan et al., 2015).

^2^
NHIS total active membership has increased since then but remains relatively low at 52% of the Ghanaian population in 2020.

^3^
In six of the intervention communities, a more intensive version of the intervention was implemented, as described in (Fenenga et al., 2018). Our results are robust to controlling for the intensity of the intervention.

^4^
The description of the intervention draws heavily on a companion paper (Alhassan, Nketiah‐Amponsah, et al., 2015) which evaluated the impact of the same intervention on medical‐technical health service quality.

^5^
Two companion papers assess the impact of the intervention on medical‐technical quality. Alhassan et al. (2015) reported that staff efforts toward increasing patient safety and risk reduction improved significantly in intervention facilities, as well as measures of accountability and staff competencies. Alhassan et al. (2019) find that the intervention facilities recorded significant improvements in maternal and child health services, most notably in terms of spontaneous vaginal deliveries at the facility, child immunizations, female condoms distribution, HIV tests for pregnant and non‐pregnant women, and malaria tests.

^6^
The US Dollar Exchange rate as at 31^st^ January 2017 was $1 = GHS₵4.2

## APPENDIX 1 Sample Size Determination

The sample size for the household survey was calculated to detect a minimum effect size of 5.0% point on active enrollment in the NHIS. Active enrollment at baseline was 0.38 (38%) on average, with a standard deviation of 0.5 (50%). The inclusion of a baseline and an endline measurement of enrollment reduces the amount of residual variation. However, correlation between the baseline and endline outcomes was unknown at the start of the study. Based on the premise that people who enroll in the current year are most likely to enroll the following year, this correlation was assumed to be relatively high and set at 0.8. Further, the random assignment of the intervention at the health facility level introduces a cluster effect, increasing the required sample size. The intra‐cluster correlation coefficient (rho) was assumed to be relatively low at 0.02. We calculated a sample of 30 households in the catchment area of each of the 64 health facilities for a total sample size of 1920 households. This will be able to detect an effect of 5.0% points at an intra‐cluster correlation of 0.02, or an effect of 10% points when the intra‐cluster correlation increases to 0.10.

Both OLS and probit regressions correcting for socio‐economic characteristics and clustering at the health facility level were done to predict the correlates of attrition. The dependent variable is a dummy which is equal to one if the individual dropped out at follow‐up and 0 if otherwise. From the analysis, receiving the engagement intervention did not significantly predict attrition, neither did any of the descriptive characteristics significantly predict attrition (Columns 1 and 3). Similarly, an interaction of individual characteristics with the treatment variable and controlling for descriptive characteristics did not significantly predict attrition (Columns 2 and 4).

**TABLE A1:** Perception Factors with their Factor Loadings and Cronbach's alpha

	Perception statements	Mean perception	Factor loading	Cronbach's alpha (α)
Healthcare quality score factors				
Complaint lodging, handling and feedback	How satisfied are you with the place/desk for lodging complaints at the facility?	2.92	0.87	0.92
	How satisfied are you with the process of lodging complaint at the facility?	2.92	0.91	
	How satisfied are you with the complaint handling and feedback by the health facility?	2.96	0.87	
Respect, compassion and friendliness of health staff	The Docs./Med. Assistants/Nurses are compassionate and very supportive	2.99	0.81	0.77
	The Docs./Med. Assistants/Nurses treat me respectfully	2.98	0.80	
	There is a well‐organized and fair queuing system	2.93	0.53	
Adequacy of information provision and service delivery	How satisfied are you with the services provided by the health facility?	2.77	0.51	0.82
	How satisfied are you with the information provided by the health facility?	2.83	0.53	
Satisfaction with waiting time	I don't have to wait for a long time to see a doctor/medical assistant	3.05	0.63	0.65
	How satisfied are you with the waiting time at the facility?	2.87	0.63	

NHIS quality score factors				
Information, service delivery and NHIS benefit	How satisfied are you with the services provided by the district NHIS office?	2.65	0.79	0.76
	How satisfied are you with the information provided by the district NHIS office?	2.69	0.78	
	How satisfied are you about the benefit you derive from the NHIS?	2.76	0.53	
ID card production and distribution	The distribution of NHIS cards is convenient	2.65	0.49	0.53
	How satisfied are you with the NHIS registration and renewal process?	2.79	0.49	
Registration fees and annual premium	The premium for the NHIS package is too high	3.07	0.79	0.85
	The registration fee is too high	3.32	0.79	
Office location and opening hours	The district scheme office location is convenient	2.87	0.62	0.66
	The district scheme office opening hours is convenient	2.76	0.62	

**TABLE A2:** Comparison of Intervention and Control Group at Baseline (balanced sample)

	Balanced sample at baseline (*N* = 5335)							
	# Obs.	Mean	Std. Err.	Intervention	Control	Difference	*p*‐value	
Panel A. Demographic characteristics								
Average age	5335	26.25	0.449	26.52	25.96	0.56	0.537	
% females	5335	54.28	0.738	54.79	53.77	1.02	0.491	
% living in rural communities	5335	53.36	6.448	55.12	51.57	3.55	0.784	
Average household size	5335	4.78	0.085	4.79	4.78	0.01	0.985	
% Christians	5335	89.47	1.037	89.35	89.59	−0.24	0.908	
Panel B. Socio‐Economic characteristics								
% primary education & above	5335	49.07	1.668	50.26	47.86	2.39	0.473	
% employed	5335	43.37	0.624	43.99	42.75	1.24	0.322	
Average annual household expenditure (GH₵)	5241	3917.10	539.73	4482.17	3348.36	1133.81	0.292	
Panel C. Healthcare utilization rate								
% Sick in last 6 months	5335	30.55	1.785	29.18	3196	−2.78	0.435	
% Visiting health facility in last 6 months	5335	39.25	2.019	39.24	39.27	−0.03	0.994	
Panel D. Insurance enrollment rate								
% currently insured	5335	41.74	1.644	44.25	39.19	5.06	0.125	
Panel E. Household Head's perception of healthcare quality								
(*N* = 1908)								
Average perception on overall healthcare quality	1502	0.005	0.029	0.012	−0.003	0.015	0.805	
Average perception on complaint lodging, handling & feedback	1463	0.002	0.039	0.033	−0.029	0.063	0.418	
Average perception on respect, compassion & friendliness	1488	0.003	0.034	−0.000	0.006	−0.006	0.921	
Average perception on information & service delivery	1475	0.004	0.029	−0.011	0.019	−0.029	0.612	
Average perception on waiting time	1412	0.009	0.041	0.027	−0.008	0.035	0.666	
Panel F. Household Head's perception of NHIS service quality (*N* = 1908)								
Average perception on overall NHIS quality	1502	−0.005	0.017	−0.027	0.018	−0.046	0.186	
Average perception on information & service provision	1483	0.001	0.034	−0.059	0.062	−0.122*	0.075	
Average perception on ID card production & waiting time	1482	0.002	0.026	−0.029	0.032	−0.061	0.237	
Average perception on registration fees & annual premium	1489	−0.022	0.029	−0.031	0.013	−0.018	0.764	
Average perception on office location & opening hours	1485	−0.001	0.032	0.007	−0.009	0.016	0.804	

*Note*: Balanced sample. The sample sizes for Panels E and F are smaller because the perception questions were posed only to household heads. *p*‐values and standard errors are robust and corrected for clustering at the health facility level.

Abbreviation: NHIS, National Health Insurance Scheme.

**p* < 0.10, ***p* < 0.05, ****p* < 0.01.

**TABLE A3:** Correlates of attrition for descriptive statistics

	OLS	OLS	Probit	Probit
	(1)	(2)	(3)	(4)
Treatment group	−0.042	0.025	−0.144	0.084
	(0.298)	(0.821)	(0.275)	(0.818)
Female	0.006	0.008	0.019	0.025
	(0.515)	(0.569)	(0.530)	(0.559)
Age	0.000	0.000	0.001	0.000
	(0.502)	(0.867)	(0.498)	(0.863)
Christian	−0.004	0.010	−0.012	0.033
	(0.923)	(0.861)	(0.920)	(0.849)
Household size	0.011	0.010	0.038	0.032
	(0.140)	(0.416)	(0.119)	(0.383)
Rural community	−0.075*	−0.046	−0.251*	−0.145
	(0.064)	(0.491)	(0.061)	(0.490)
Primary education and above	−0.005	0.010	−0.020	0.031
	(0.707)	(0.647)	(0.674)	(0.648)
Worked	−0.017	−0.009	−0.056	−0.028
	(0.252)	(0.679)	(0.250)	(0.676)
Annual household income	0.000	0.000	0.000	0.000
	(0.939)	(0.951)	(0.913)	(0.954)
Treatment group * female		−0.004		−0.014
		(0.826)		(0.814)
Treatment group * age		0.000		0.001
		(0.698)		(0.676)
Treatment group * Christian		−0.034		−0.124
		(0.655)		(0.621)
Treatment group * household size		0.002		0.011
		(0.875)		(0.817)
Treatment group * rural community		−0.062		−0.239
		(0.435)		(0.364)
Treatment group * primary education and above		−0.031		−0.109
		(0.272)		(0.244)
Intervention * worked		−0.017		−0.064
		(0.552)		(0.499)
Treatment group * annual household income		−0.000		−0.000
		(0.959)		(0.968)
Constant	0.236***	0.207***	−0.725***	−0.815***
	(0.000)	(0.007)	(0.000)	(0.001)
N	6803	6803	6803	6803

*Note*: Full (unbalanced) sample. OLS (columns 1–2) and probit (columns (3–4) estimations. Standard errors are in parentheses, robust and corrected for clustering at the health facility level.

Abbreviation: OLS, ordinary least square.

**p* < 0.10, ***p* < 0.05, ****p* < 0.01.

**TABLE A4:** Impact of community engagement (CE) on household heads' perception of healthcare quality (full sample, no controls)

	Overall healthcare quality	Complaint lodging & feedback	Respect & friendliness of staff	Information & service delivery	Waiting time
	(1)	(2)	(3)	(4)	(5)
Year	0.024	0.038	0.020	0.023	0.014
	(0.074)	(0.098)	(0.078)	(0.071)	(0.092)
Treatment group	0.019	0.051	0.004	−0.007	0.029
	(0.047)	(0.068)	(0.053)	(0.051)	(0.061)
Treatment group * year (= impact of CE)	−0.039	−0.073	−0.029	−0.032	−0.024
	(0.095)	(0.126)	(0.102)	(0.097)	(0.129)
Controls	No	No	No	No	No
Strata fixed effects	Yes	Yes	Yes	Yes	Yes
Observations	3515	3456	3493	3475	3397

*Note*: Based on the full (unbalanced) sample. Estimates are based on OLS regressions. Standard errors are in parentheses, robust and corrected for clustering at the health facility level. All equations include randomization strata fixed effects.

Abbreviations: CE, Community Engagement intervention; OLS, ordinary least square.

**p* < 0.10, ***p* < 0.05, ****p* < 0.01.

**TABLE A5:** Impact of community engagement (CE) on household heads' perception of healthcare quality (balanced sample, with controls)

	Overall healthcare quality	Complaint lodging & feedback	Respect & friendliness of staff	Information & service delivery	Waiting time
	(1)	(2)	(3)	(4)	(5)
Year	0.013	0.026	0.020	0.001	0.005
	(0.080)	(0.103)	(0.083)	(0.079)	(0.101)
Treatment group	0.016	0.063	−0.006	−0.023	0.033
	(0.052)	(0.076)	(0.057)	(0.055)	(0.069)
Treatment group * year (= impact of CE)	−0.026	−0.059	−0.031	−0.002	−0.016
	(0.103)	(0.135)	(0.110)	(0.104)	(0.138)
Controls	Yes	Yes	Yes	Yes	Yes
Strata fixed effects	Yes	Yes	Yes	Yes	Yes
Observations	2916	2877	2902	2889	2826

*Note*: Based on the balanced sample. Estimates are based on OLS regressions. Standard errors are in parentheses, robust and corrected for clustering at the health facility level. All equations include randomization strata fixed effects and sex, religion, household size, employment status, educational level as control variables.

Abbreviations: CE, Community Engagement intervention; OLS, ordinary least square.

**p* < 0.10, ***p* < 0.05, ****p* < 0.01.

**TABLE A6:** Impact of community engagement (CE) on household heads' perception of national health insurance scheme (NHIS) service quality (full sample, no controls)

	Overall insurance services quality	Information & service delivery	ID card production & distribution	Registration fees & annual premium	Office location & opening hours
	(1)	(2)	(3)	(4)	(5)
Year	0.000	0.007	0.029	−0.043	0.006
	(0.055)	(0.093)	(0.084)	(0.044)	(0.074)
Treatment group	−0.045	−0.098	−0.064	−0.041	0.019
	(0.036)	(0.065)	(0.050)	(0.051)	(0.062)
Treatment group * year (= impact of CE)	0.006	−0.005	−0.051	0.091	−0.007
	(0.074)	(0.123)	(0.108)	(0.064)	(0.114)
Controls	No	No	No	No	No
Strata fixed effects	Yes	Yes	Yes	Yes	Yes
N	3515	3489	3487	3494	3490

*Note*: Based on the full (unbalanced) sample. Estimates are based on OLS regressions. Standard errors are in parentheses, robust and corrected for clustering at the health facility level. All equations include randomization strata fixed effects and sex, religion, household size, employment status, educational level as control variables.

Abbreviations: CE, Community Engagement intervention; OLS, ordinary least square.

**p* < 0.10, ***p* < 0.05, ****p* < 0.01.

**TABLE A7:** Impact of community engagement (CE) on household heads' perception of national health insurance scheme (NHIS) service quality (balanced sample, with controls)

	Overall insurance services quality	Information & service delivery	ID card production & distribution	Registration fees & annual premium	Office location & opening hours
	(1)	(2)	(3)	(4)	(5)
Year	−0.018	−0.040	−0.004	−0.005	−0.022
	(0.053)	(0.093)	(0.083)	(0.042)	(0.075)
Treatment group	−0.045	−0.121*	−0.062	−0.015	0.012
	(0.038)	(0.067)	(0.052)	(0.054)	(0.063)
Treatment group * year (= impact of CE)	0.021	0.045	−0.028	0.050	0.023
	(0.075)	(0.125)	(0.108)	(0.065)	(0.119)
Controls	Yes	Yes	Yes	Yes	Yes
Strata fixed effects	Yes	Yes	Yes	Yes	Yes
N	2916	2897	2896	2903	2899

*Note*: Based on the balanced sample. Estimates are based on OLS regressions. Standard errors are in parentheses, robust and corrected for clustering at the health facility level. All equations include randomization strata fixed effects and sex, religion, household size, employment status, educational level as control variables.

Abbreviations: CE, Community Engagement intervention; OLS, ordinary least square.

**p* < 0.10, ***p* < 0.05, ****p* < 0.01.

**TABLE A8:** Impact of community engagement (CE) on frequency of illness and health facility visits (full sample, no controls)

	Frequency of illness		Frequency of health facility visits	
	Extensive margin	Intensive margin	Extensive margin	Intensive margin
	Logit	Truncated Poisson model	Logit	Truncated Poisson model
	(1)	(2)	(3)	(4)
Year	1.343***	0.150	−0.356***	0.720***
	(0.115)	(0.163)	(0.099)	(0.216)
Treatment group	−0.080	−0.104	0.117	−0.368
	(0.111)	(0.167)	(0.099)	(0.261)
Treatment group * year (= impact of CE)	−0.357**	0.029	−0.348**	0.495*
	(0.175)	(0.182)	(0.157)	(0.289)
Controls	No	No	No	No
Strata fixed effects	Yes	Yes	Yes	Yes
*N*	13,071	4590	13,071	3646
Marginal effect	−0.084**	0.012	−0.060**	0.295
	(0.041)	(0.076)	(0.027)	(0.183)

*Note*: Based on the full (unbalanced) sample. Estimates are based on a hurdle model with a logit in the first stage (extensive margin) and a truncated Poisson model in the second stage (intensive margin). Standard errors are in parentheses, robust and corrected for clustering at the health facility level. All equations include randomization strata fixed effects.

Abbreviation: CE, Community Engagement intervention.

**p* < 0.10, ***p* < 0.05, ****p* < 0.01.

**TABLE A9:** Impact of community engagement (CE) on frequency of illness and health facility visits (balanced sample, with controls)

	Frequency of illness		Frequency of health facility visits	
	Extensive margin	Intensive margin	Extensive margin	Intensive margin
	Logit	Truncated Poisson model	Logit	Truncated Poisson model
	(1)	(2)	(3)	(4)
Year	1.553***	0.122	−0.258**	0.609***
	(0.134)	(0.191)	(0.112)	(0.223)
Treatment group	−0.087	−0.084	0.133	−0.389
	(0.133)	(0.180)	(0.108)	(0.263)
Treatment group * year (= impact of CE)	−0.319	−0.048	−0.353**	0.526*
	(0.208)	(0.204)	(0.161)	(0.278)
Controls	Yes	Yes	Yes	Yes
Strata fixed effects	Yes	Yes	Yes	Yes
*N*	10,483	3703	10,483	2939
Marginal effect	−0.069	−0.020	−0.061**	0.321*
	(0.045)	(0.084)	(0.028)	(0.182)

*Note*: Based on the balanced sample. Estimates are based on a hurdle model with a logit in the first stage (extensive margin) and a truncated Poisson model in the second stage (intensive margin). Standard errors are in parentheses, robust and corrected for clustering at the health facility level. All equations include randomization strata fixed effects and sex, religion, household size, employment status, educational level as control variables.

Abbreviation: CE, Community Engagement intervention.

**p* < 0.10, ***p* < 0.05, ****p* < 0.01.

**TABLE A10:** Impact of community engagement (CE) on insurance enrollment (no controls)

Dependent variable	Insured	Insured	Insured at endline	Insured at endline
	Unbalanced sample	Balanced sample	Balanced sample	Balanced sample
			Uninsured at baseline	Insured at baseline
Sample	(1)	(2)	(3)	(4)
Year	−0.056	−0.093		
	(0.071)	(0.073)		
Treatment group	0.118*	0.129*		
	(0.063)	(0.066)		
Impact of CE (treatment group * year)	0.092	0.132	0.340***	0.203*
	(0.088)	(0.089)	(0.111)	(0.116)
Controls	No	No	No	No
Strata fixed effects	Yes	Yes	Yes	Yes
N	13,071	10,483	2923	2112
Marginal effect	0.035	0.050	0.106***	0.074*
	(0.033)	(0.034)	(0.034)	(0.042)

*Note*: Column 1 is based on the full (unbalanced) sample, Column 2 on the balanced sample. Columns 3 and 4 are based the balanced sample, for the subsets of households that were uninsured and insured, respectively, at baseline. Estimates are based on probit models. Columns 3 and 4 use only endline data in the estimation and estimate insurance status at endline as a function of treatment. Standard errors are in parentheses, robust and corrected for clustering at the health facility level. All equations include randomization strata fixed effects.

Abbreviation: CE, Community Engagement intervention.

**p* < 0.10, ***p* < 0.05, ****p* < 0.01.

**TABLE A11:** Impact of community engagement (CE) on frequency of illness and health facility visits (full sample, with controls, households not insured at baseline)

	Frequency of illness		Frequency of health facility visits	
	Extensive margin	Intensive margin	Extensive margin	Intensive margin
	Logit	Truncated Poisson model	Logit	Truncated Poisson model
	(1)	(2)	(3)	(4)
Year	1.716***	0.048	−0.107	0.460
	(0.148)	(0.222)	(0.131)	(0.383)
Treatment group	−0.079	0.010	0.136	−0.218
	(0.148)	(0.220)	(0.115)	(0.402)
Treatment group * year (= impact of CE)	−0.384*	−0.201	−0.382*	0.106
	(0.232)	(0.265)	(0.200)	(0.461)
Controls	Yes	Yes	Yes	Yes
Strata fixed effects	Yes	Yes	Yes	Yes
*N*	6948	2280	6948	1745
Marginal effect	−0.082*	−0.066	−0.064*	0.045
	(0.049)	(0.087)	(0.033)	(0.198)

*Note*: Based on the full (unbalanced) sample of households that were not insured at baseline. Estimates are based on a hurdle model with a logit in the first stage (extensive margin) and a truncated Poisson model in the second stage (intensive margin). Standard errors are in parentheses, robust and corrected for clustering at the health facility level. All equations include randomization strata fixed effects and sex, religion, household size, employment status, educational level as control variables.

Abbreviation: CE, Community Engagement intervention.

**p* < 0.10, ***p* < 0.05, ****p* < 0.01.

**TABLE A12:** Impact of community engagement (CE) on frequency of illness and health facility visits (full sample, with controls, households insured at baseline)

	Frequency of illness		Frequency of health facility visits	
	Extensive margin	Intensive margin	Extensive margin	Intensive margin
	Logit	Truncated Poisson model	Logit	Truncated Poisson model
	(1)	(2)	(3)	(4)
Year	1.513***	0.270	−0.549***	0.896***
	(0.188)	(0.208)	(0.113)	(0.186)
Treatment group	−0.048	−0.204	0.051	−0.580**
	(0.142)	(0.205)	(0.122)	(0.252)
Treatment group * year (= impact of CE)	−0.331	0.137	−0.247	0.946**
	(0.269)	(0.250)	(0.177)	(0.379)
Controls	Yes	Yes	Yes	Yes
Strata fixed effects	Yes	Yes	Yes	Yes
*N*	5024	1809	5024	1703
Marginal effect	−0.069	0.073	−0.045	0.818*
	(0.056)	(0.134)	(0.032)	(0.454)

*Note*: Based on the full (unbalanced) sample of households that were insured at baseline. Estimates are based on a hurdle model with a logit in the first stage (extensive margin) and a truncated Poisson model in the second stage (intensive margin). Standard errors are in parentheses, robust and corrected for clustering at the health facility level. All equations include randomization strata fixed effects and sex, religion, household size, employment status, educational level as control variables.

Abbreviation: CE, Community Engagement intervention.

**p* < 0.10, ***p* < 0.05, ****p* < 0.01.
